# SPG4 and Dementia: Expanding the Clinical Spectrum

**DOI:** 10.1002/acn3.70371

**Published:** 2026-03-26

**Authors:** Emanuele Panza, Arun Meyyazhagan, Eliseo Picchi, Gustavo Ribas, Ingrid Faber, Ryosuke Miyamoto, Preethi Basavaraju, Paolo Eusebi, Haripriya Kuchi Bhotla, Mario Stasi, Fabrizio Gaudiello, Francesco Patti, Filippo Maria Santorelli, Marcondes Cavalcante França, José Luiz Pedroso, Orlando Graziani Povoas Barsottini, Hélio Afonso Ghizoni Teive, Peter Henry St George‐Hyslop, Toshitaka Kawarai, Antonio Orlacchio

**Affiliations:** ^1^ Department of Medical and Surgical Sciences University of Bologna Bologna Italy; ^2^ Medical Genetics Unit IRCCS Azienda Ospedaliero‐Universitaria di Bologna Bologna Italy; ^3^ Department of Medicine and Surgery University of Perugia Perugia Italy; ^4^ Department of Biomedicine and Prevention University of Rome “Tor Vergata” Rome Italy; ^5^ Department of Internal Medicine Federal University of Paraná (UFPR) Curitiba PR Brazil; ^6^ Department of Neurology University of Brasília Brasília DF Brazil; ^7^ Department of Neurology Tokushima University Graduate School of Biomedical Sciences Tokushima Japan; ^8^ Department of Human Genetics and Molecular Biology Bharathiar University Coimbatore TN India; ^9^ Department of Diagnostic Imaging Guidonia Radiology Studio Rome Italy; ^10^ Department G.F. Ingrassia University of Catania Catania Italy; ^11^ Department of Neurobiology and Molecular Medicine IRCCS Stella Maris Foundation Pisa Italy; ^12^ Department of Neurology University of Campinas (UNICAMP) Campinas SP Brazil; ^13^ Department of Neurology Federal University of São Paulo (UNIFESP) São Paulo SP Brazil; ^14^ Department of Neurology, Taub Institute for Research on Alzheimer's Disease and the Aging Brain Columbia University Irving Medical Center New York New York USA; ^15^ Department of Neurology Hyogo Prefectural Harima‐Himeji General Medical Center Himeji Japan; ^16^ European Centre for Brain Research (CERC) IRCCS Santa Lucia Foundation Rome Italy

**Keywords:** Alzheimer's disease, dementia, hereditary spastic paraplegia, SPG4, thin *corpus callosum*

## Abstract

**Objective:**

Hereditary spastic paraplegia (HSP) is a group of disorders characterized by progressive spasticity and lower limb weakness, with mutations in SPG4/*SPAST* being the most common cause. Detailed studies and clinical and molecular comparisons across different populations are missing. We examined the clinical, pathological, and genetic spectrum of the SPG4/*SPAST* gene in patients with HSP.

**Methods:**

The study involved 726 HSP patients recruited from Italy, Brazil, and Japan between 2001 and 2025, with analysis conducted in collaborative centers. SPG4/*SPAST* variants were identified using direct and next‐generation sequencing. The pathogenicity of novel variants was confirmed through familial segregation and in silico analysis.

**Results:**

Clinical and epidemiological differences were observed across populations, particularly in phenotype, age at onset, and disability, expanding the SPG4 clinical spectrum. Genetic analysis identified 52 pathogenic SPG4/*SPAST* mutations in 284 patients, including four novel variants. Several mutations were population‐specific, and a possible founder effect was suggested for a recurrent variant in Italy. Dementia occurred in 44 HSP‐SPG4 patients and was neuropathologically confirmed in four unrelated autopsied cases from four families comprising 28 individuals; atypical pathological features were observed in all four autopsied cases. Additionally, 18 patients with SPG4/*SPAST* mutations presented with thin *corpus callosum* and intellectual disability.

**Interpretation:**

In this study, we investigated pathogenic SPG4/*SPAST* variants in an international cohort of HSP patients from three continents. Our findings expand the clinical spectrum of HSP‐SPG4, identifying a new type complicated by an atypical pathological form of dementia. Careful assessment of genotype–phenotype relationships offers insights into patient counseling and future research planning.

## 1 Introduction

Hereditary spastic paraplegias (HSPs) are a heterogeneous group of clinically and genetically inherited neurological disorders with the main characteristic being the degeneration of the central nervous system leading to weakness of the lower limbs and spastic paraparesis, which can progress and eventually cause difficulties in walking [1, 2]. When the clinical manifestations of spastic paraparesis are essentially characterized by pyramidal signs, the disease is classified as *pure*. On the other hand, we define *complicated* forms where spastic paraparesis is accompanied by other neurological or non‐neurological features. For instance, the HSP clinical picture can be complicated by neurological signs such as cognitive impairment, thin *corpus callosum* (TCC), intellectual disability, cerebellar atrophy, polyneuropathy, epilepsy, but also non‐neurological features such as skeletal anomalies, skin, or connective tissue alterations, amyotrophy, optic atrophy, and ocular abnormalities [3, 4].

To date, more than 87 distinct loci have been reported to be associated with the disease, and 73 causative genes have been cloned [5]. The onset of HSP can vary from young to patients older than 70 years old [3]. HSP can be inherited as autosomal dominant (AD), autosomal recessive (AR), X‐linked (XL), or mitochondrial (MT) conditions, with AD forms being the most common among them [6].

Despite the observed genetic heterogeneity, mutations in the SPG4*/SPAST* gene (MIM604277) are the single most common cause identified in AD‐HSP cases [7, 8]. SPG4 (MIM182601) most often presents as pure form, with an average of symptoms typically starting between the second and the fourth decade of life. Other common features that complicate the clinical picture of different SPG4 patients are *pes cavus*, hearing impairment [9], scoliosis [10], and/or cognitive dysfunction [11, 12]. The SPG4/*SPAST* gene encodes for spastin. This protein belongs to the ATPases Associated with diverse cellular Activities family (AAA). The most characterized role of spastin is linked to membrane trafficking and in the regulation of cellular microtubules reorganization [6]. More than 300 different pathogenic variants in the SPG4/*SPAST* gene have been described and linked to HSP [3].

In this study, we set out to investigate the SPG4/*SPAST* mutation profile in an international HSP cohort of patients from Italy, Brazil, and Japan, to identify novel pathogenetic variants in SPG4/*SPAST* and to correlate these variants to the associated clinical presentation.

## 2 Materials and Methods

### 2.1 Patient Cohort

Between 2001 and 2025, 726 patients with a diagnosis of HSP were recruited. Of these, 628 were familial and 98 sporadic cases. Among the familial cases, 313 were probands (172 Italian, 93 Brazilian, and 48 Japanese) diagnosed with pure or complicated HSP.

Most Italian patients came from southern and central Italy (120; 70%), while 52 (30%) were from northern regions. Italian origin was established by family name and place of birth. The Brazilian cohort included individuals of Native Brazilian, African, European, and Arab ancestry. Japanese patients were mainly from the Kansai region or Shikoku Island.

Clinical data were collected, and neurological examinations were performed by movement disorders specialists. Disease onset was defined as gait disturbance. Severity and progression were assessed using the Spastic Paraplegia Rating Scale (SPRS) [13]. All patients showed typical HSP features with corticospinal tract involvement (progressive lower limb spasticity, weakness, and extensor plantar response). Patients with early and prominent spastic paraplegia consistent with a degenerative disorder and no alternative causes were included. Those with vitamin deficiency, HTLV‐1 infection, or structural brain lesions were excluded.

A positive family history with autosomal dominant inheritance was documented in 215 affected individuals (121 Italian, 56 Brazilian, 38 Japanese). Ninety‐eight patients (51 Italian, 37 Brazilian, 10 Japanese) were classified as sporadic due to negative or unavailable family history. When probands carried SPG4/*SPAST* mutations, available affected relatives were also analyzed. Control samples were obtained from 200 Italian, 87 Brazilian, and 109 Japanese healthy volunteers.

Clinical diagnosis of dementia was based on DSM‐5 or DSM‐5‐TR criteria together with NINCDS‐ADRDA Alzheimer's criteria [14, 15, 16]. Intellectual disability was diagnosed according to DSM‐5/DSM‐5‐TR criteria [14, 15]. Cognitive assessment in patients older than 20 years included the MMSE and CAMCOG [17, 18], while the WISC‐IV or WISC‐V was used in individuals aged 6–16 years [19, 20]. In patients with dementia, CSF Aβ42/Aβ40 ratio and p‐tau181 levels were measured to assess Alzheimer's disease core biomarkers [21].

Ethical approval is reported in the online Supporting Information file.

### 2.2 Genetic Analysis

Genomic DNA from patients and controls was isolated from whole blood using Maxwell 16 System (Promega, Madison, WI, USA). Once extracted, the DNA has been amplified and the SPG4/*SPAST* gene was investigated by Sanger Sequencing analyzing the entire coding regions composed of the 17 exons and at least 30 bp of flanking intronic sequences (ABI PRISM 3130xl Genetic Analyzer, Applied Biosystems, Waltham, MA, USA).

Whole‐Exome Sequencing (WES) or Whole‐Genome Sequencing (WGS) was also performed to confirm the exclusive pathological change in *SPAST* (NOVASEQ 6000, Illumina, San Diego, CA, USA). WGS was executed in HSP patients with cognitive decline or intellectual disability.

Multiplex Ligation‐dependent Probe Amplification (MLPA) (P165‐C1 HSP, MRC‐Holland) has been used to detect large genomic rearrangements involving SPG4*/SPAST* in patients with negative results of Sanger analysis and WES or WGS.

Segregation analyses were performed by using a PCR‐Restriction Fragment Length Polymorphism method (PCR‐RFLP) (data accessible on demand) or through Sanger Sequencing. Haplotype analysis was performed on the seven Italian families where a novel SPG4/*SPAST* variant was identified and in one Brazilian and two Japanese kindred in which patients harbor a same deletion mutation (microsatellite markers D2S365, D2S390, D2S352, D2S2347, D2S367, and D2S2230).

Supporting Information file details information of genetic study.

### 2.3 Statistical Analysis

Descriptive statistics was computed for all variables and reported as medians, range, and InterQuartile Range (IQR) for continuous variables or frequencies and percentages for categorical variables. The Pearson's chi‐squared and Fisher's exact test, when appropriate, were used to detect between groups differences in the distribution of categorical variables. The Mann–Whitney and the Kruskal‐Wallis tests, when appropriate, were used to detect between groups differences in the distribution of continuous variables.

A multivariate logistic regression model was developed using stepwise regression (forward selection) with predictive variables that were significant in the univariate analyses, to evaluate the association between presence of specific clinical features and mutation status. The enter and remove limits were *p* = 0.10 and *p* = 0.15, respectively. The statistical software package used for all the analyses was the Statistical Package for the Social Sciences (SPSS) for Windows (version 21.0; SPSS Inc., Chicago IL, USA).

### 2.4 Pathological Studies

Autopsy was performed on four patients with HSP‐SPG4 and cognitive impairment and was not available for the remaining cases, mainly due to lack of consent or because death occurred outside our pathological referral network. Standard post‐mortem neuropathological evaluation was carried out on 5‐μm sections of the proband's brain across multiple cortical and subcortical regions, including the frontal, temporal, and parietal cortices; posterior cortical areas (precuneus and posterior cingulate gyrus); hippocampus; entorhinal cortex; amygdala; thalamus; striatum; and substantia nigra. Histological staining of paraffin‐embedded material was performed using hematoxylin and eosin or Congo red.

Immunohistochemical detection was performed using β‐amyloid (βA4) and glial fibrillary acidic protein (GFAP) antibodies. Images were acquired at 20× and 40× magnification. Immunofluorescence double staining using α‐synuclein and phosphor‐TDP‐43 antibodies was performed to assess colocalization of the two proteins.

### 2.5 Neuroimaging Investigations

Complete neuroimaging studies were carried out in all patients with HSP‐SPG4 and cognitive disfunction or intellectual disability. Brain and spinal cord MRI were performed by routinely used T1, T2, as well as two‐dimensional (2D) fluid attenuated inversion recovery (FLAIR) sequences, and three‐dimensional (3D) FLAIR sequence was added when needed. A reliable protocol was employed to perform amyloid PET imaging in selected individuals with HSP‐SPG4 and associated cognitive dysfunction.

## 3 Results

### 3.1 Characteristics of the HSP Patient's Cohort

In this study, 726 patients were enrolled from three populations. They comprised 383 males (52.8%) and 343 females (47.2%) with no significant differences in gender distribution (Tables 1 and 2).

**TABLE 1:** Comparison of SPG4 mutation carriers and noncarriers.

	SPG4 positive	SPG4 negative	TOT	*p*
	*n* = 284 (%)	*n* = 442 (%)	726	
Sex				
Male	153 (53.9)	230 (52.0)	383	0.63
Female	131 (46.1)	212 (48.0)	343	
Age at onset, years				
Median	40.5	23.0	28.0	< 0.0001
IQR range	25.0–50.2	16.0–34.0	18.0–43.0	
Range	1–69	2–69	1–69	
Disease duration, years				
Median	12.0	8.0	9.0	< 0.0001
IQR range	6.0–18.0	5.0–12.0	6.0–14.0	
Range	1–40	1–31	1–40	
Type				
Pure	212 (74.6)	156 (35.3)	368	< 0.0001
Complicated	72 (25.4)	286 (64.7)	358	
Disability stage				
1	81 (28.5)	171 (38.7)	252	< 0.0001
2	71 (25.0)	134 (30.3)	205	
3	60 (21.1)	85 (19.2)	145	
4	42 (14.8)	37 (8.4)	79	
5	30 (10.6)	15 (3.4)	45	
LL hypereflexia	284 (100)	442 (100)	726	NA
LL spasticity	278 (97.9)	435 (98.4)	713	0.96
LL weakness	244 (85.9)	383 (86.7)	627	0.94
Babinsky sign	276 (97.2)	431 (97.6)	707	0.98
Unilateral	11 (4)	9 (2.1)	20	0.14
Bilateral	265 (96)	422 (97.9)	687	
Sphincter disturbances	154 (54.2)	215 (48.6)	369	0.40
Standardized cognitive testing	244 (85.9)	376 (85.1)	620	0.835
MMSE, score				
Median	27	27	27	0.222
IQR range	26–29	26–28	26–28	
Range	5–30	1–30	1–30	
CAMCOG score				
Median	89	89	89	0.222
IQR range	86–96	86–92	86–92	
Range	16–99	3–99	3–99	

*Note:* Significant at *p* < 0.05.

Abbreviations: IQR, inter quartile range; LL, lower limbs; NA, not applicable.

**TABLE 2:** Comparison of clinical features of SPG4 positive and negative patients within the three populations.

	SPG4 positive			*p*	SPG4 negative			*p*
	*n* = 284 (%)				*n* = 442 (%)			
	Italian	Brazilian	Japanese		Italian	Brazilian	Japanese	
	*n* = 156	*n* = 76	*n* = 52		*n* = 247	*n* = 124	*n* = 71	
Sex								
Male	81 (51.9)	39 (51.3)	28 (53.8)	0.96	126 (51.0)	65 (52.4)	35 (49.3)	0.91
Female	75 (48.1)	37 (48.7)	24 (46.2)		121 (49.0)	59 (47.6)	36 (50.7)	
Age at onset, years								
Median	34.0	43.0	41.5	< 0.001	23.0	27.0	21.0	< 0.0001
IQR range	17.0–47.0	34.8–55.0	30.0–52.0		13.5–31.0	21.0–37.2	15.0–28.5	
Range	1–67	16–69	2–67		2–69	7–69	4–53	
Disease duration, years								
Median	12.0	16.0	7.0	< 0.001	7.0	8.0	9.0	0.085
IQR range	7.0–18.5	9.0–23.5	5.0–9.0		5.0–12.0	5.0–12.0	6.0–14.0	
Range	1–35	1–40	1–22		1–28	1–31	1–22	
Type								
Pure	122 (78.2)	39 (51.3)	41 (78.8)	< 0.0001	152 (61.5)	38 (30.6)	23 (32.4)	< 0.0001
Complicated	34 (21.8)	37 (48.7)	11 (21.2)		95 (38.5)	86 (69.4)	48 (67.6)	
Disability stage								
1	43 (27.6)	7 (9.2)	23 (44.3)	< 0.0001	97 (39.3)	18 (14.5)	28 (39.4)	< 0.0001
2	42 (26.9)	12 (15.8)	18 (34.6)		69 (27.9)	21 (16.9)	24 (33.8)	
3	40 (25.6)	20 (26.3)	9 (17.3)		52 (21.1)	23 (18.6)	11 (15.5)	
4	23 (14.7)	20 (26.3)	1 (1.9)		21 (8.5)	40 (32.4)	6 (8.5)	
5	8 (5.2)	17 (22.4)	1 (1.9)		8 (3.2)	22 (17.6)	2 (2.8)	
LL hyperreflexia	156 (100)	76 (100)	52 (100)	NA	247 (100)	124 (100)	71 (100)	NA
LL spasticity	152 (97.4)	75 (98.6)	51 (98.1)	0.99	243 (98.4)	122 (98.4)	70 (98.6)	0.99
LL weakness	135 (86.5)	67 (88.5)	42 (80.7)	0.94	219 (88.7)	110 (88.7)	54 (76.1)	0.74
Babinsky sign	152 (97.4)	74 (97.4)	50 (96.2)	0.99	238 (96.4)	123 (99.2)	70 (98.6)	0.98
Unilateral	7 (4.6)	3 (4.1)	1 (2)	0.72	6 (2.5)	2 (0.8)	1 (1.4)	0.78
Bilateral	145 (96.4)	71 (95.9)	49 (98)		232 (97.5)	121 (99.2)	69 (98.6)	
Sphincter disturbances	82 (52.54)	47 (61.8)	25 (48)	0.67	130 (52.6)	56 (45.1)	40 (56.3)	0.64
Standardized cognitive testing	133 (85.2)	68 (89.5)	43 (82.7)	0.523	210 (85.0)	106 (85.5)	60 (84.5)	0.983
MMSE, score								
Median	27	27	27	0.629	89	89	92	0.302
IQR range	26–29	20–29	27–29		86–92	86–92	83–96	
Range	5–30	6–30	5–30		3–99	36–99	36–99	
CAMCOG score								
Median	89	89	89	0.629	89	89	92	0.303
IQR range	86–96	65–96	89–94		86–92	86–92	83–96	
Range	16–99	20–99	16–99		3–99	36–99	36–99	

*Note:* Significant at *p* < 0.05.

Abbreviations: IQR, InterQuartile Range; LL, lower limbs; NA, not applicable.

### 3.2 Genetic Analysis

In our cohort, 60.9% of patients did not present pathogenetic variants in *SPAST*, including 365 ad‐HSP familial affected subjects and 77 sporadic HSP cases. Direct sequencing of SPG4*/SPAST* gene revealed 52 different heterozygous mutations in 263 ad‐HSP and 21 sporadic cases (Table S1). A total of 156 patients were Italian, 76 were from Brazil, and 52 were Japanese. We found 20 missense mutations (39.2%), eight splice‐site mutations (15.7%), seven small deletions (four frameshift and three in‐frame) (13.7%), five nonsense mutations (9.8%), two small insertions (all frameshift) (3.9%), one insertion/deletion (2.0%), and one no‐sense change (2.0%). Eight different large rearrangements (15.7%), including six multi‐exon deletions, one complete deletion, and one duplication were identified by MLPA analyses. Patients carrying missense mutations showed a lower median age at onset (36.0 years) compared with those carrying other mutational classes (42.0 years).

**FIGURE 1:**
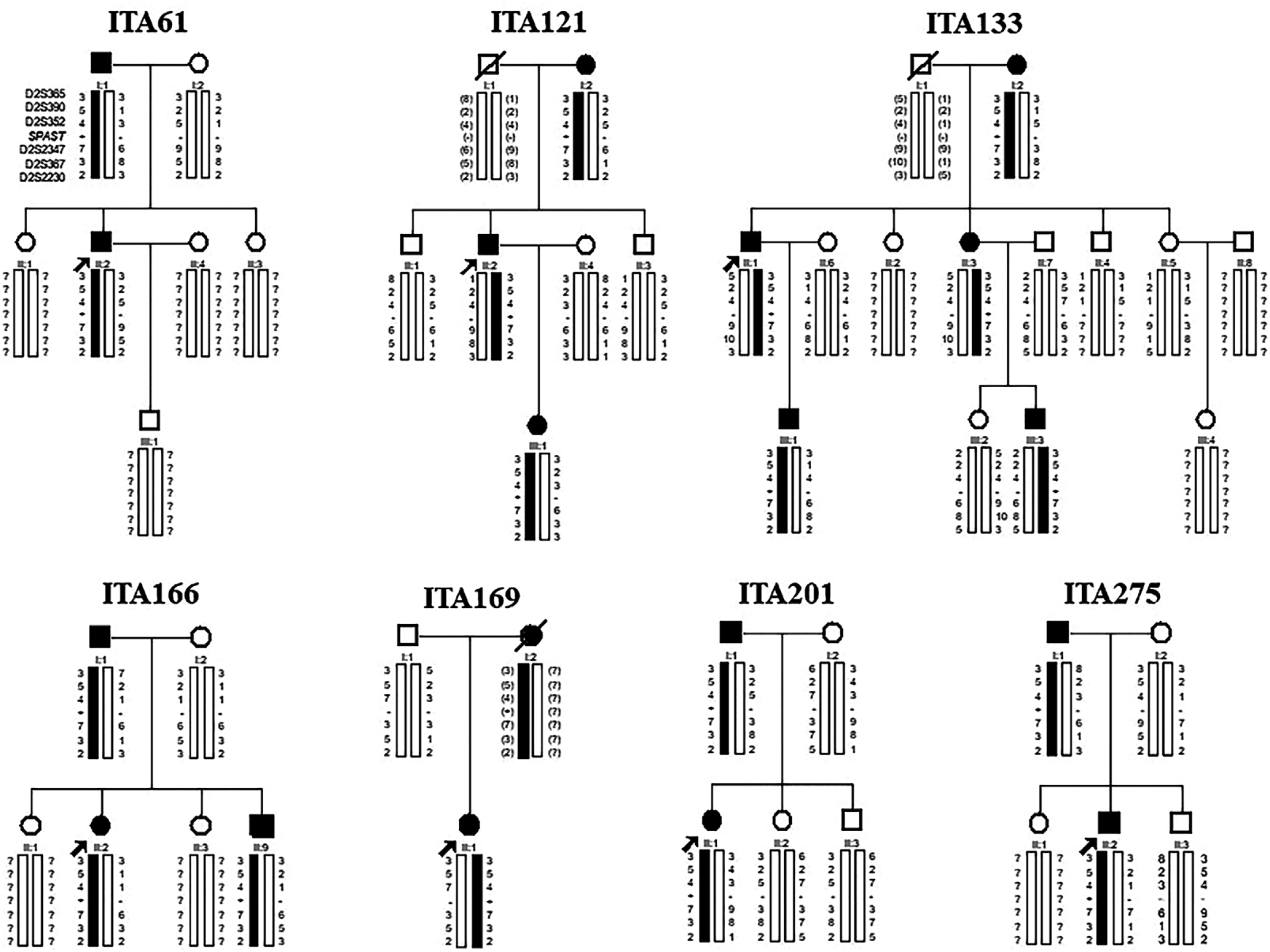
Pedigrees and segregation chart of seven SPG4 families carrying the same novel variant c.1382T>G (p.Leu461Arg) and sharing the same haplotype. Black solid symbols indicate affected individuals carrying the mutation; white symbols represent unaffected subjects; square symbols are men, circles are women, and slashed symbols are deceased individuals. Individuals are reported as code numbers below the symbols. Probands are marked by arrows. Haplotypes are depicted with markers D2S365, D2S390, D2S352, D2S2347, D2S367, and D2S2230 from top to bottom. Reconstructed genotypes are indicated by parentheses and question marks symbolize alleles that could not be reconstructed. Explanation is needed for + and − mark.

Through analyzing the recent literature and genome databases we could establish that, among all, four novel variants were identified, including two missense mutations, one small deletion, and one deletion/insertion (Table S1). The variants are not reported in the GnomAD databases (https://gnomad.broadinstitute.org) and absent in 200 Italian (female 52.5%, median age 46.2 years, IQR: 30–62), 87 Brazilian (female 49%, median age 38.8 years, IQR: 26–59), and 109 Japanese controls (female 56.0%, median age 61.8 years, IQR: 44–79). The variants co‐segregated with the disease in all familial pedigrees and the pathogenetic effect of the coding variant was predicted (Table S2). In particular, the novel variant c.1382T>G (p.Leu461Arg) was found in 18 unrelated patients from seven Italian families and the same small deletion of exon 13 (c.1534_1536del) was detected in 10 unrelated patients from one Brazilian as well as two Japanese pedigrees. We found a common haplotype in all the Italian patients harboring the novel variant c.1382 T>G (p.Leu461Arg) (Figure 1), whereas a similar analysis did not reveal a common haplotype in 10 carriers (five Brazilian and five Japanese) with the same small deletion of exon 13 (c.1534_1536del). All 284 affected individuals carrying SPG4/*SPAST* mutations were also analyzed by WES. Examination of coding regions and adjacent splice sites revealed a single pathogenic variant in the *SPAST* gene, whereas all other variants detected in different genes were classified as nonpathogenic or of uncertain significance. Overall, these results support *SPAST* as the primary genetic contributor to the disorder, consistent with HSP.

Besides, we were able to perform a genetic diagnosis in 96/313 families (31.0%) and 15/96 SPG‐positive kindred (15.6%) were with two affected. Furthermore, a total of 21/98 sporadic cases (21.4%) were positive. In this category, a considerable difference between Italian (24%), Brazilian (16%), and Japanese (30%) cases was observed, being the Japanese population the highest.

Finally, in our study, affected members of the SPG4 families did not exhibit anticipation, as reported before [9, 22, 23, 24].

### 3.3 Phenotypic Spectrum

We compared the clinical characteristics of SPG4 mutation carriers with those of noncarriers using univariate analysis (Tables 1 and 2). In this cohort, SPG4 patients showed a significantly later age at onset and a longer disease duration compared with noncarriers (*p <* 0.0001). Moreover, the clinical phenotype was predominantly pure (*p <* 0.0001) and, according to the subdivision into five stages of disability based on the SPRS scale (1 = 1–10; 2 = 11–20; 3 = 21–31; 4 = 32–41; 5 = 42–52) [13], SPG4 patients exhibited a lower degree of disability (*p <* 0.0001) (Tables 1 and 2). Among the different populations, Brazilian patients showed the highest median age at onset (43.0 years), compared with Italian (34.0 years) and Japanese patients (41.5 years). The phenotype is more often pure in Italian and Japanese HSP‐SPG4 patients than in Brazilian (*p <* 0.0001). Japanese SPG4 patients exhibited significantly lower disability levels compared with all other groups, predominantly corresponding to disability stages 1 and 2 (*p* < 0.0001) related to a shorter disease duration (*p* < 0.0001). In contrast, patients carrying SPG4 mutations from Italy and Brazil showed comparable disability profiles and were mainly classified within disability stages 4 and 5, indicating more severe impairment (*p* < 0.0001) associated with a longer disease duration (*p* < 0.0001) (Table 2). Refinement of these results by multivariate analysis confirmed that a higher rate of SPG4/*SPAST* mutations was significantly associated with (i) median age at onset (*p* < 0.0001, OR: 0.49; 95% CI: 0.38–0.64), (ii), phenotype (*p* < 0.0001, OR: 6.42; 95% CI: 4.37–9.43), and (iii) disability stage (*p* < 0.006, OR: 1.27; 95% CI: 1.07–1.50), which remain the unique features independently predicting a mutated SPG4/*SPAST*. Furthermore, familial setting was found statistically associated to the mutation status (*p* < 0.0001, OR: 4.63; 95% CI: 3.30–6.51).

Clinical evaluation of SPG4/*SPAST* patients with complicated disease (*n* = 72) showed that foot deformities, such as *pes cavus* or *pes equinus*, and cognitive deficits were the main complications (50/72 and 44/72; 69.4% and 61.1%, respectively). The number of patients who underwent standardized cognitive testing (MMSE and CAMCOG), as well as their scores, are reported in Tables 1 and 2.

**FIGURE 2:**
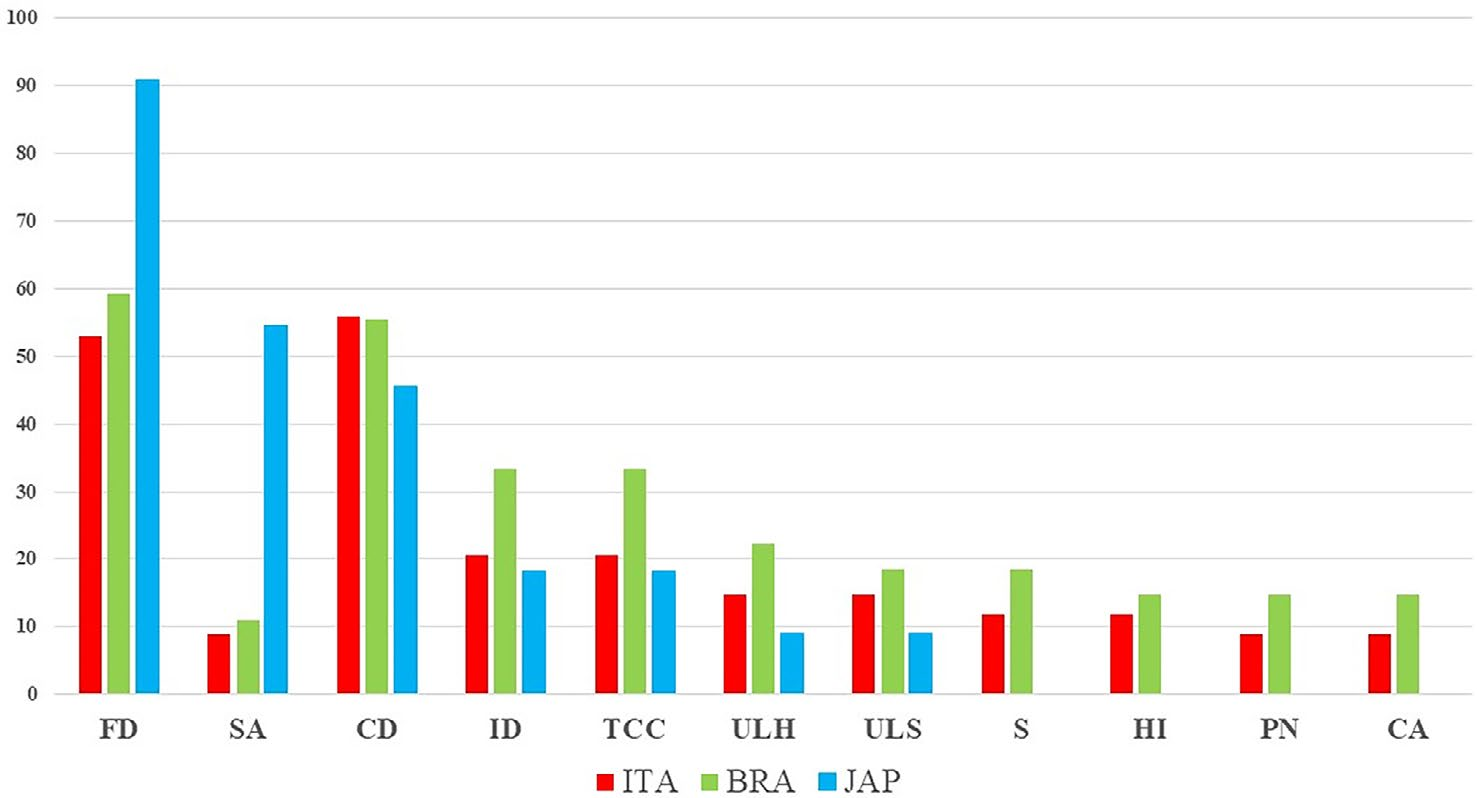
Complicated SPG4‐positive phenotypes. Percentage of SPG4‐positive patients showing complicated phenotypes in the Italian (Red), Brazilian (green), and Japanese (Blue) populations. CA, cerebellar atrophy; CD, cognitive dysfunctions; FD, foot deformities; HI, hearing impairment; ID, intellectual disability; PN, peripheral neuropathy; S, seizures; SA, skeletal abnormalities; TCC, thin *corpus callosum*; ULH, upper limb hyperreflexia; ULS, upper limb spasticity.

Furthermore, a wide variation of phenotypic expression was detected within patients among the three populations. Japanese HSP complicated patients showed mainly foot deformities and skeletal abnormalities, for instance scoliosis or vertebral anomalies, respect complicated affected individuals of the Italian and Brazilian populations. Moreover, Japanese HSP complicated patients displayed less cognitive impairment, intellectual disability, or cerebral defects, such as TCC, respect to Italian and Brazilian complicated subjects. No seizures, hearing impairment, cerebellar atrophy, and peripheral neuropathy were reported in Japanese patients (Figure 2).

Remarkably, all HSP Italian patients carrying the new mutation c.1382T>G (p.Leu461Arg) (18 affected from seven families) had features compatible with a diagnosis of early onset dementia. We also evaluated one Brazilian kindred (BRA‐36; five patients) and two Japanese pedigrees (JAP‐28; JAP‐31; respectively, three and two affected individuals) in which the SPG4/*SPAST* small deletion of exon 13 (c.1534_1536del) co‐segregate with HSP and early onset dementia. In all family members diagnosed with early‐onset dementia, including those without neuropathological confirmation, the CSF Aβ_42_/Aβ_40_ ratio was decreased, consistent with cerebral Aβ_42_ plaque accumulation. In contrast, CSF p‐tau181 levels were within normal or low ranges, suggesting the absence of significant tau pathology or neurofibrillary tangle formation.

Lastly, of the 18 patients with TCC in addition to intellectual disability, two patients were Japanese, seven were Italian, and nine were Brazilian.

Clinical data for the representative Italian family (ITA‐133), including one autopsy‐confirmed case, are shown in Table S3. Autopsy‐confirmed emblematic cases from the Brazilian (BRA‐36 II:1) and Japanese families (JAP‐28 II:1; JAP‐31 II:3) are shown in Table S4. Clinical data for the representative Brazilian family (BRA‐56), including six patients with TCC and intellectual disability, are shown in Table S5.

### 3.4 Pathological Assessments

**FIGURE 3:**
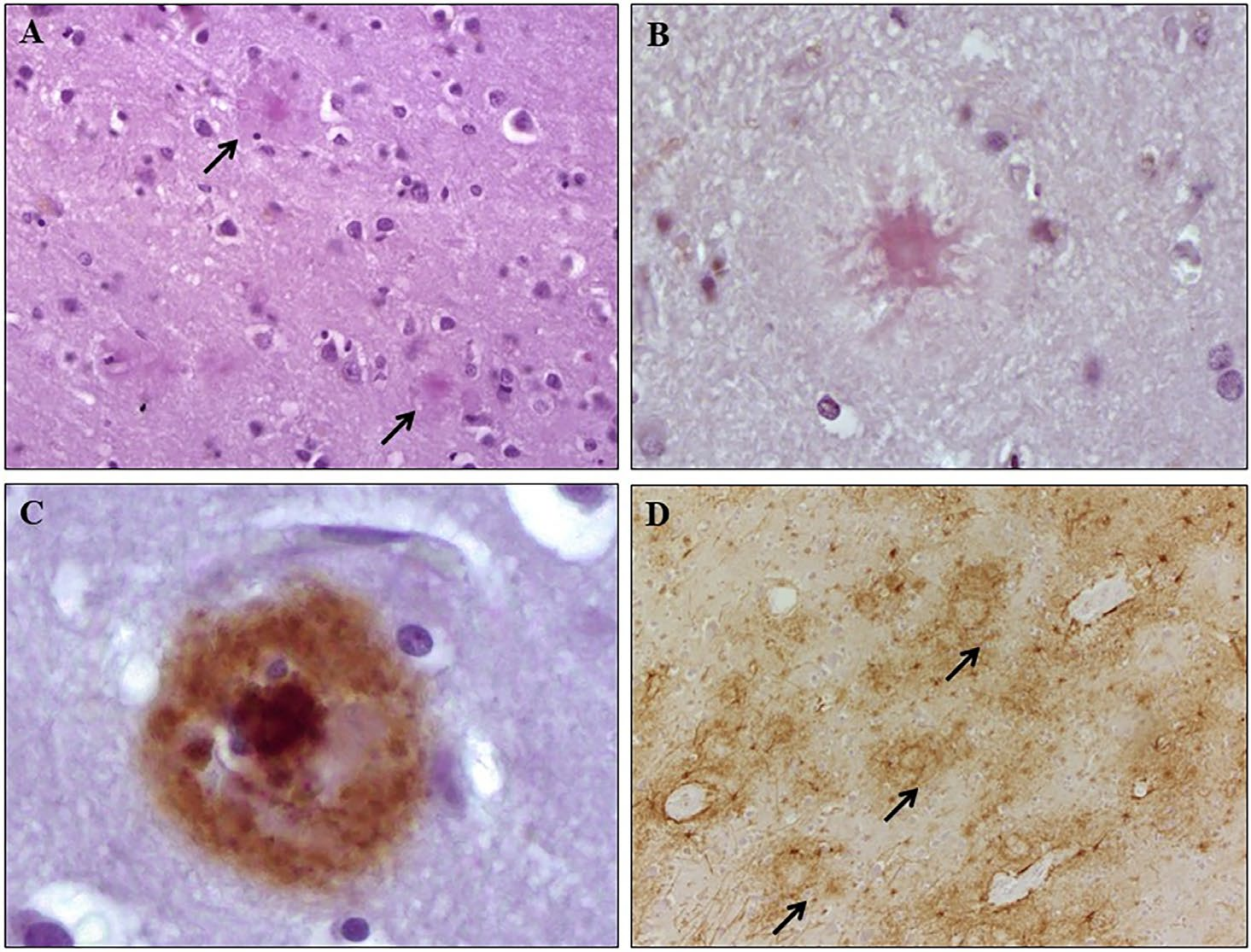
Pathology of dementia with amyloid plaques. Family ITA133—Patient II:1. Senile plaques in frontal cortex section (A, arrows). Standard coloration with hematoxylin and eosin (A), as well as congo red (B). Immunohistochemical detection of β Amyloid antibody (βA4) (C) and Glial Fibrillary Acidic Protein (GFAP) antibody coloring to highlight the high astrocytosis, particularly evident around vases and plaques (D, arrows). Magnification: 20× (A, D); 40× (B, C).

A post‐mortem brain examination was performed on patient II:1 (aged 64 at death and at autopsy) from Family ITA133, who carried the novel SPG4/*SPAST* pathogenic mutation c.1382T>G. The brain (865 g) had noticeable atrophy of the frontal, temporal, and parietal lobes. The caudate nucleus was atrophic (Grade IV), and there was medium ventricular dilatation. Posterior cortical areas, such as the precuneus and posterior cingulate gyrus, were also atrophic. The hippocampus, amygdala, as well as the entorhinal cortex, were harshly atrophic. Senile plaques with a congophilic core and neuritic pathology were abundant in all cortical areas, amygdala, hippocampus, thalamus, substantia nigra, as well as striatum, and cotton wool plaques (CWP), burnt‐out plaques, non‐cored neuritic plaques, diffuse plaques, neurofibrillary tangles, as well as Lewy bodies were absent. Immunofluorescence double‐staining with the combination of α‐synuclein and phospho‐TDP‐43 did not detect the presence of abnormal aggregates of the proteins. Prion diseases were excluded at autopsy. Precisely, the case was classified as plaque‐only dementia (POD) and fulfills criteria for A3‐B0‐C3 scores in the National Institute on Aging‐Alzheimer's Association (NIA‐AA) ABC scoring system for evaluating Alzheimer's disease neuropathologic change [25, 26]. The density of neuritic plaques was classified as “frequent,” in accordance with the CERAD criteria [27]. Immunohistochemical analysis showed elevated astrocytosis remarkably apparent around vases and plaques (Figure 3). Reliable with a diagnosis of HSP, the spinal cord was thinner than typical and compressed in its anteroposterior dimension. Mild degeneration of the corticospinal tract began at the pons position and became moderate–severe at the spinal cord level.

Comparable neuropathological findings, satisfying A3‐B0‐C3 criteria according to the NIA‐AA ABC scoring system and categorized as “frequent” neuritic plaque density based on CERAD criteria, were identified in another Italian patient with cognitive impairment carrying the novel SPG4/*SPAST* pathogenic variant c.1382T>G (individual II:2, Family ITA166; onset at age 45, death and autopsy at age 76), as well as in two additional patients: one Brazilian (onset at 43, death at 79, autopsy at 80) and one Japanese (onset at 51, death and autopsy at 82), both exhibiting cognitive impairment and harboring the pathogenic SPG4/*SPAST* exon 13 microdeletion (c.1534_1536del).

All autopsy‐confirmed cases were homozygous for the apolipoprotein E (APOE) ε4 allele (ε4/ε4). Among patients presenting with cognitive impairment, 66.7% were homozygous (APOE ε4/ε4) and 33.3% were heterozygous (APOE ε3/ε4).

### 3.5 Neuroimaging Evaluations

**FIGURE 4:**
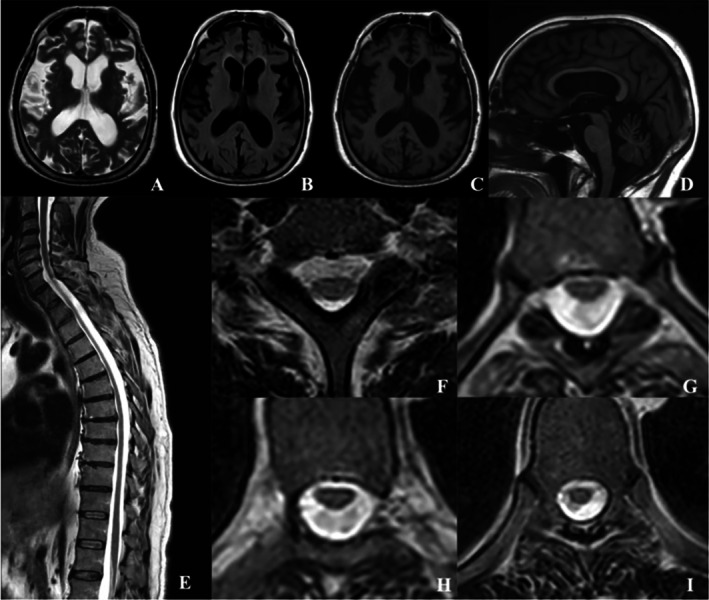
Neuroimaging assessment. Brain and spine MRIs. Family ITA201—Patient II:1. Neuroimaging findings of dementia. Axial spin‐echo (SE) T2‐weighted (A), axial Fluid Attenuated Inversion Recovery (FLAIR) T2‐weighted (B), and axial SE T1‐weighted (C) show severe atrophy of the temporal lobes and enlargement of the Sylvian fissures. Index Brazilian Patient with intellectual disability. (D) Midline sagittal SE T1‐weighted MRI scan showing generalized thinning of the *corpus callosum*, without focal lesions. Index Japanese Patient. MRI of the spine. (E) Sagittal Turbo SE (TSE) T2‐weighted shows widespread thinning of the spinal cord. Axial TSE T2‐weighted at C7 (F), D5 (**G**), D7 (H), and D9 (I) level confirmed the reduction in thickness of the spinal cord without abnormal medullary signal intensity.

Brain MRI in HSP‐SPG4 patients with cognitive issues showed cerebral atrophy (Figure 4A–C). Those with intellectual disability had TCC without cerebellar involvement or white matter abnormalities, including the “Ears of the Lynx” (Figure 4D). In all these subjects, the spinal cord MRI revealed no signs of cord compression or abnormal cord signal, with thinning of the spinal cord, especially in the cervical and thoracic regions. There was no correlation between the degree of spinal cord atrophy and duration as well as severity of the disease (Figure 4E–I).

**FIGURE 5:**
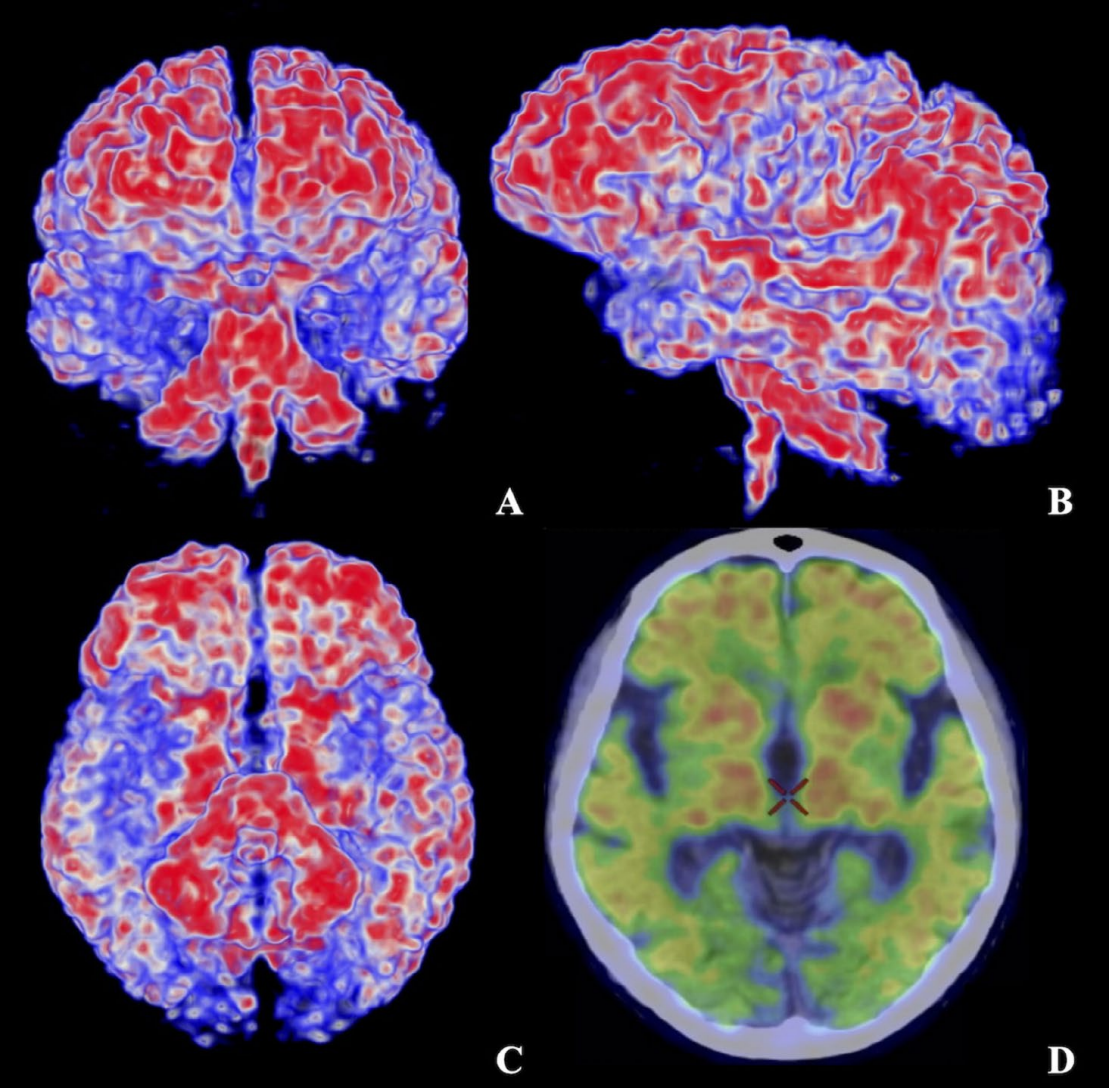
Neuroimaging assessment. Amyloid PET brain imaging. Family ITA275—Patient II:2. (A–C) Three‐Dimensional (3D) volume renderings of an ^18^F‐flutemetamol PET brain scan, illustrating regional cortical tracer binding indicative of cerebral β‐amyloid deposition. The color map reflects tracer retention: High retention (red) suggests a significant burden of amyloid plaques, while low retention (blue) indicates negative or nonspecific binding. (D) Axial slice from the same ^18^F‐flutemetamol PET scan, showing radiotracer distribution across cortical and subcortical brain regions. Warmer colors (red/yellow) represent areas of higher tracer uptake, whereas cooler tones (blue/green) indicate lower uptake. The image demonstrates relatively symmetrical cortical tracer retention, particularly in the frontal and parietal lobes, suggestive of significant cerebral β‐amyloid deposition.

In all patients with early‐onset dementia from the four families that included an individual with autopsy‐confirmed diagnosis, ^18^F‐flutemetamol PET scans revealed cortical tracer binding consistent with manifest cerebral β‐amyloid deposition (Centiloid scores > 50), even in the absence of overt neuropathological findings (Figure 5).

## 4 Discussion

We conducted a genetic‐epidemiological study of 726 HSP patients from Italy, Brazil, and Japan, identifying 52 SPG4/*SPAST* mutations. Our findings expand the understanding of SPG4/*SPAST* in HSP and suggest that two novel mutations could be linked to a new form of HSP associated with a pathological subtype of dementia with amyloid plaques.

In our cohort, 60.9% of unrelated individuals lacked pathogenic *SPAST* variants, including 365 ad‐HSP familial subjects and 77 sporadic cases. These patients had a lower median age at onset, a more complicated phenotype, and higher disability. Further investigation of mutations in other genes using NGS technologies like RNA sequencing or WGS is recommended.

**FIGURE 6:**
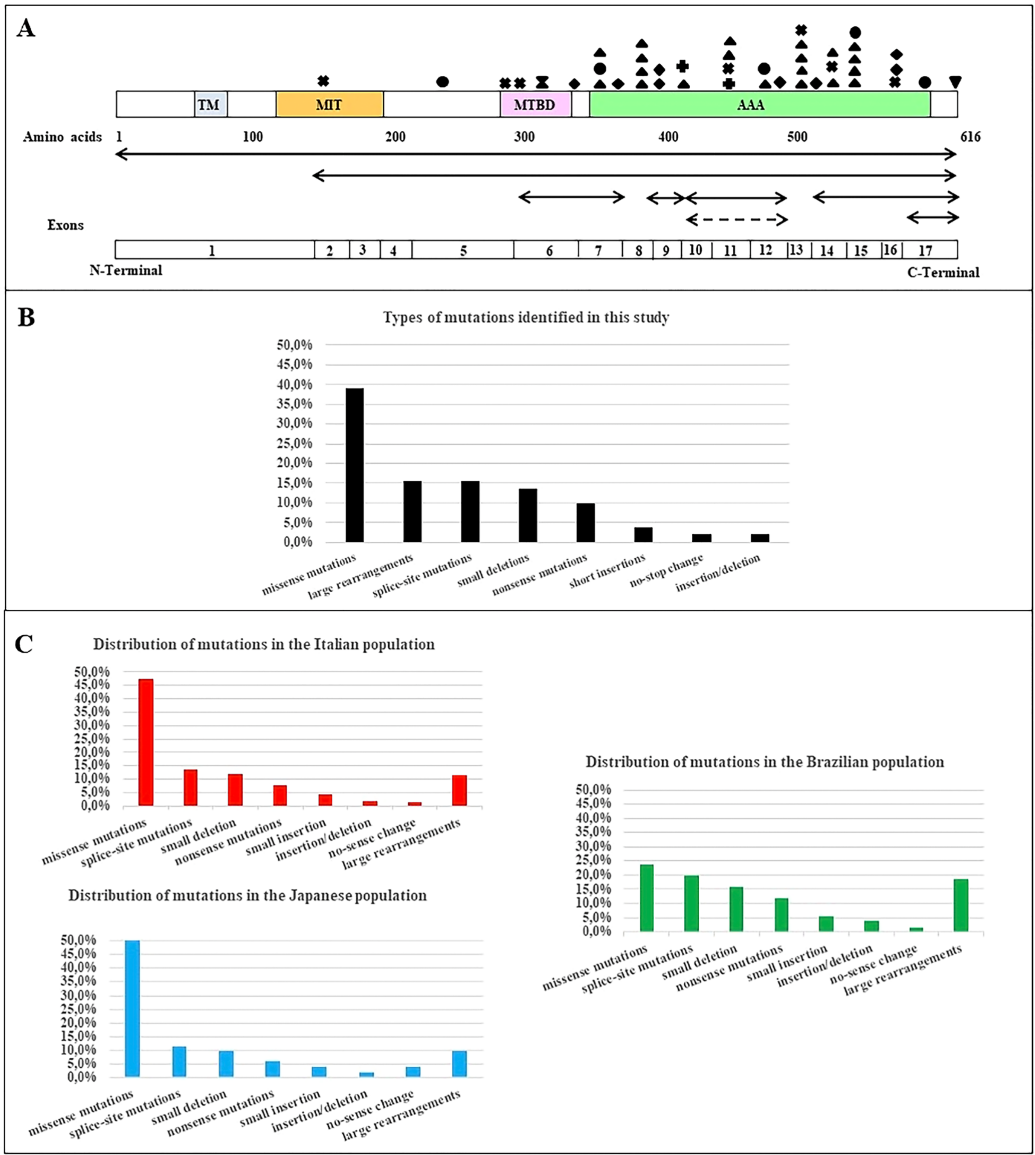
Graphic depiction of SPG4/*SPAST* gene and protein domains with mutations and charts showing the relative frequencies of diverse types of variants. (A) Schematic representation of SPG4/*SPAST* gene and protein domains with mutations identified in this study. Black triangle symbol, missense mutation; black rhombus symbol, splice‐site mutation; black x symbol, small deletion; black circle symbol, nonsense mutation; black cross symbol, small insertion; black hourglass symbol, indel; black inverted triangle symbol, no‐stop change; line with arrow both ends, large deletion; dotted line with arrow both ends, large duplication. (B) the chart shows the relative frequencies of distinct kinds of mutations. (C) the charts indicate the relative frequencies of different types of mutations within the three populations. Red, Italian; Green, Brazilian; Blue, Japanese.

In all three populations, most mutations cluster within the AAA domain, without specific *hot spots*. Of the 52 mutations, 39.2% are missense mutations, likely producing stable spastin proteins with a dominant‐negative effect [12]. The remaining mutations are truncating, likely degraded by nonsense‐mediated mRNA decay (Figure 6) [28].

Missense variants represented the most prevalent mutational class among Italian and Japanese patients, accounting for 47.4% and 53.9% of the identified genetic defects, respectively. In contrast, Brazilian patients more frequently carried variants leading to pre‐terminal stop codons (57.9%), which were observed more often than missense mutations (23.7%) (Figure 6). This distribution may contribute to the earlier age at onset observed in Italian and Japanese patients, in line with previous reports [29].

Among all the pathogenetic variants, the percentage of large deletions/duplications (15.7%) is comparable in Italian (11.6%) and Brazilian patients (18.4%), while it is lower in Japanese patients (9.7%). These observations are consistent with the previous SPG4 series worldwide, with relative frequencies ranging from 3.8% to 37.5% in the largest cohorts [30]. Their presence reaffirms prior evidence that the Alu genomic architecture of SPG4/*SPAST* predisposes to diverse genomic rearrangements, leading to haploinsufficiency [31]. Moreover, it confirms that gene rearrangements should be examined routinely in patients who are clinically diagnosed with HSP.

The most frequent mutation in our cases was the novel c.1382T>G (p.Leu461Arg) variant in the AAA domain, found in 18 Italian subjects. All affected individuals shared a common haplotype, suggesting a possible founder effect (Figure 1). In contrast, no common haplotype was found in the 10 patients with the exon 10–12 deletion.

Comparison by feature between SPG4/*SPAST* mutation carriers and noncarriers revealed statistically significant results for (i) median age at onset, (ii) phenotype, and (iii) disability stage (Table 1). Similar results were detected in all the populations analysed and are consistent with previous studies from the three different countries [11, 30, 32, 33, 34, 35, 36].

Over a quarter (25.4%) of SPG4 patients had a complicated phenotype, with foot deformities being the most frequent (Figure 2 and Table 1). These abnormalities did not affect life expectancy, but the complex phenotype often included cognitive issues such as intellectual disability and a diagnosis of dementia with amyloid pathology.

The association of spastic paraparesis and dementia has already been widely described. Some patients with PSEN1 pathogenetic variants display an uncharacteristic clinical manifestation characterized by spastic paraplegia preceding Alzheimer's disease [37, 38, 39, 40, 41]. In Alzheimer's disease, brain tissue analysis typically shows the presence of congophilic amyloid plaques with neuritic pathology and neurofibrillary tangles as a typical hallmark [39], although in the subjects affected by spastic paraplegia preceding Alzheimer's disease, the brain histopathology is distinct, and it is characterized by diffuse CWP without a congophilic core and with only minor neuritic dystrophy [37, 38, 39, 40, 41]. Moreover, neurofibrillary tangles represent a defining feature of Alzheimer's pathology, whereas Lewy bodies are frequently observed as a co‐pathology in individuals with *PSEN1*‐associated Alzheimer's disease [38, 40, 41, 42, 43], but in our cases CWP, neurofibrillary tangles, as well as Lewy bodies were absent, and the patients had only senile plaques associated with marked astrocytosis. The originality of POD has already been reported in rare forms of Alzheimer's disease or in unusual types of dementia with cortical Lewy bodies [43, 44, 45, 46], and the POD cases are older at the disease onset and death and seem to have a shorter disease duration and slower cognitive deterioration [45]. Interestingly, our SPG4 cases with partial Alzheimer's type pathology at autopsy do not share those features, and the findings indicate that they might somewhat match variants of HSP for an unknown pathogenic mechanism of action. Additionally, all autopsy‐confirmed cases were homozygous for the APOE ε4 allele, which may be related to the observed pathology, as the ε4/ε4 genotype promotes a biological environment highly permissive to the development of amyloid‐rich, plaque‐dominated dementia [47]. Furthermore, it is hypothesized that patients without neuropathological confirmation were also affected by POD, as CSF biomarker profiles in addition to amyloid PET analysis indicated the presence of amyloid pathology in the absence of tau‐related neurodegeneration. Specifically, these individuals exhibited a decreased Aβ_42_/Aβ_40_ ratio alongside normal or low p‐tau181 levels, suggestive of amyloid plaque deposition without concomitant neurofibrillary tangle formation. Moreover, the long disease duration observed in many patients with cognitive deficits (Table S3) makes it unlikely that the absence of tau tangles can be attributed to age‐related factors.

The clinical evaluation suggests that these cases involve more than a simple cognitive disorder, with histopathological analysis supporting a diagnosis of dementia with amyloid pathology. Since WES and WGS did not identify mutations in other genes, especially those linked to Alzheimer's disease, we propose that these SPG4 variants may be associated with a new disease manifestation, potentially identifying a form of HSP linked to an atypical pathological type of dementia.

A previous post‐mortem study in SPG4 patients found novel hyaline inclusions in anterior horn cells and changes in cytoskeletal protein and mitochondrial immunostaining. These findings suggest that long tract degeneration is linked to cytopathology in the motor system, possibly indicating a disruption in cytoskeletal function [48]. Additionally, although senile plaques were absent, the study showed tau pathology beyond the motor system, suggesting that spastin‐related HSP neuropathology extends beyond the motor system, like our results.

Similar findings have also been reported in association with *SPAST* exon 17 deletion, showing widespread ubiquitin‐positive inclusions in the absence of significant amyloid deposition and further supporting the heterogeneity of dementia‐related changes associated with *SPAST* mutations [49].

Another feature identified was TCC with intellectual disability, highly suggestive of SPG11 and SPG15 forms [50, 51, 52, 53, 54], present in 18/72 complicated patients (25.0%), mainly Brazilian. WES and WGS in affected subjects did not identify pathogenic variants in additional genes, suggesting these mutations may be linked to HSP‐TCC. This sign is occasional in SPG4 patients [9]; thus, the high TCC plus intellectual disability rate supports further investigation of SPG4/*SPAST* mutations to clarify mechanisms of maldevelopment or neurodegeneration [55].

The “Ears of the Lynx” is an imaging sign that indicates degeneration of the forceps minor, usually evaluated by FLAIR MRI [56]. While it can resemble acquired conditions such as ependymitis granularis, it is a hallmark of certain HSPs, particularly types SPG11 and SPG15. However, other types of HSPs have also been reported to exhibit this sign, especially when accompanied by atrophy of the anterior portion of the *corpus callosum* [57]. In our cases, we did not observe this sign despite the presence of TCC.

Comparing the cohorts, it was found that Japanese patients had fewer and less severe signs and symptoms compared to Brazilian and Italian patients. This could be explained by the longer disease duration of the Brazilian and Italian affected subjects. The similarity between Brazilian and Italian cohorts may stem from the massive Italian immigration to Brazil in different historical periods. Nevertheless, further epidemiological studies are needed to examine the broad range of factors that might explain ethnic differences.

Our molecular analysis identified mutations in 31% of the cohort. This frequency is in the range of previous studies analyzing SPG4/*SPAST* mutations in Italy (21%–45%) [58], in Brazil (up to 37%) [35], and in Japan (26%) [59].

A total of 21% of sporadic cases were positive, hypothesizing occurrence of *de novo* SPG4/*SPAST* mutations or low/altered penetrance SPG4/*SPAST* mutations in these patients. Our results in Italian (24%) and Japanese (30%) sporadic patients appear significantly higher than previous studies (6%–20%) [58, 59], while prior data on Brazilian populations are unspecified. Surprisingly, the likelihood of mutations in sporadic cases (21%) is higher than in families with two affected (16%), suggesting that the number of sporadic cases is overestimated, which include not only *de novo* mutations but also incomplete penetrance cases, nonpaternity, or inexact clinical assessment of the parents. Therefore, the effective number of familial cases with two affected may be higher than observed. These results suggest that the classification in sporadic HSP cases is a restrictive concept since the dichotomy between familial and sporadic HSP patients is less clear than previously assumed. However, further confirmations are needed before translating our preliminary results into the clinical setting.

The limitation of our study lies in using varied sequencing technologies due to the extensive duration of the research and limited WGS for SPG4‐negative patients, except for those with dementia with amyloid plaques and TCC, which may have prevented the detection of new genetic changes. Additionally, to confirm the founder hypothesis for the SPG4/*SPAST* mutation c.1382 T>G (p.Leu461Arg), a larger haplotype analysis and detailed archival research on Church and Municipal Registries should be conducted to validate the results obtained from the laboratory study.

In summary, this study extends the mutational, pathological, and clinical spectrum of HSP‐SPG4 and provides evidence for a new form of HSP complicated by a pathological variant of dementia with amyloid plaques. Taking together, our findings suggest that SPG4 mutations may predispose carriers to an increased vulnerability to early‐onset dementia. Further investigations are required to determine the link between the mutated spastin and the pathological hallmarks of Alzheimer's disease and to clarify the underlying mechanisms of this association.

## Author Contributions

**Emanuele Panza:** drafting/revision of the manuscript for content, including medical writing for content; study concept or design; analysis or interpretation of data. **Arun Meyyazhagan:** drafting/revision of the manuscript for content, including medical writing for content; major role in the acquisition of data; and analysis or interpretation of data. **Eliseo Picchi:** drafting/revision of the manuscript for content, including medical writing for content; major role in the acquisition of data. **Gustavo Ribas:** major role in the acquisition of data. **Ingrid Faber:** major role in the acquisition of data. **Ryosuke Miyamoto:** major role in the acquisition of data. **Preethi Basavaraju:** major role in the acquisition of data. **Paolo Eusebi:** analysis or interpretation of data. **Haripriya Kuchi Bhotla:** major role in the acquisition of data. **Mario Stasi:** major role in the acquisition of data. **Fabrizio Gaudiello:** major role in the acquisition of data. **Francesco Patti:** major role in the acquisition of data. **Filippo Maria Santorelli:** major role in the acquisition of data. **Marcondes Cavalcante França Jr.:** major role in the acquisition of data. **José Luiz Pedroso:** major role in the acquisition of data. **Orlando Graziani Povoas Barsottini:** major role in the acquisition of data. **Hélio Afonso Ghizoni Teive:** major role in the acquisition of data. **Peter Henry St George‐Hyslop:** major role in the acquisition of data. **Toshitaka Kawarai:** drafting/revision of the manuscript for content, including medical writing for content; major role in the acquisition of data; and analysis or interpretation of data. **Antonio Orlacchio:** drafting/revision of the manuscript for content, including medical writing for content; major role in the acquisition of data; study concept or design; and analysis or interpretation of data.

## Funding

This work was supported by a grant from the Italian Ministry of University and Research (*Piano Nazionale di Ripresa e Resilienza*, grant no. CN00000041 to E.Pa.), grants from the Italian Ministry of Health (grants n. RC2025 as well as RC5x1000 to F.M.S. and grant no. RF19.12 to A.O.), grants from the Department of Medicine and Surgery of the University of Perugia (*Fondo Ricerca di Base*, grants no. DSCH_BASE19_ORLACCHIO and RICERCABASE_2020_ORLACCHIO to A.O.), and a grant from the University of Perugia (*Progetto di Ricerca di Ateneo*, grant no. RICERCA_ATENEO_ALIMENTAZIONE to A.O.). The funders played no role in the study design, data collection, analysis, data interpretation, or the writing of this manuscript.

## Conflicts of Interest

The authors declare no conflicts of interest.

## Supporting information


**Table S1:** Variants identified in this study.
**Table S2:** Bioinformatic analysis of the new variants identified.
**Table S3:** Clinical findings of the patients of family ITA‐133.
**Table S4:** Clinical findings of the index patients of families BRA‐36, JAP‐28, and JAP‐31.
**Table S5:** Clinical findings of the patients of family BRA‐56.

## Data Availability

Data sets are not freely available due to ethical and privacy restrictions. Requests should be directed at the corresponding author.
