# Supplementary material for: SPG4 and Dementia: Expanding the Clinical Spectrum
Source: Ann Clin Transl Neurol. 2026 Mar 26:10.1002/acn3.70371. Online ahead of print. doi: 10.1002/acn3.70371 (PMC13394015; doi:10.1002/acn3.70371)
Supplement: Supplementary file 1 — Table S1: Variants identified in this study. Table S2: Bioinformatic analysis of the new variants identified. Table S3: Clinical findings of the patients of family ITA‐133. Table S4: Clinical findings of the index patients of families BRA‐36, JAP‐28, and JAP‐31. Table S5: Clinical findings of the patients of family BRA‐56. [file ACN3-9999-0-s001.doc]

**Supplemental Materials**

**Supplementary Materials and Methods**

**Patient Cohort** *-* **Ethical Approval**

The study was performed according to a protocol reviewed and approved by the Ethics Committee of the University of Perugia, Perugia, Italy, in addition to the IRCCS Santa Lucia Foundation, Rome, Italy, as well as local institutional review boards of the referring institutions. The subjects’ consent was acquired agreeing to the Declaration of Helsinki (<https://www.wma.net/policies-post/wma-declaration-of-helsinki/>).

**Genetic Analysis - Extended Information**

*Direct sequencing*

Oligonucleotide primers (data available upon request) have been designed by the software Primer Blast (<https://www.ncbi.nlm.nih.gov/tools/primer-blast/>). Electropherograms were analysed and compared to the reference sequences (GenBank accession number: NM_014946.3).

*Next-generation sequencing (NGS)*

The WES analysis was operated by the SeqCap EZ Choice Enrichment Kits (Hoffmann-La Roche, Basel, Switzerland). All coding exons of the RefSeq transcripts of the genes and 15 base pairs of the flanking introns were targeted. 99% of the coding exons were sequenced by a minimal read depth of 30X. WGS libraries were organized, and sequencing was completed by 2X150 bp cycles. Sequence runs were measured, and only high-quality FASTQ data (exhibiting a 99.9% base call accuracy) were managed. Direct assessment of data sequence was operated by the Integrative Genomics Viewer v.2.3. A second examination through GenomeUp platform was carried out (<https://lab.juliaomix.com/>) managing the Best Practices Workflows of Genome Analysis ToolKit v4.1 (GATK) for germline variant calling.

*MLPA investigation*

For each sample, relative peak areas were calculated and compared with three wild type controls (Coffalyser 7.0, MRC-Holland, Amsterdam, Netherlands).

*Bioinformatics*

DNA changes were categorised with the assistance of public databases (GnomAD, [https://gnomad.broadinstitute.org](https://gnomad.broadinstitute.org/)) and in agreement with the American College of Medical Genetics Guideline for germline variant classification [pathogenic (class 5), probable pathogenic (class 4), and Variants of Undefined Significance (VoUS; class 3)] [S1]. *In silico* analyses were carried out by PolyPhen 2, Mutation Taster, and Sorting Intolerant From Tolerant (SIFT) software.

*Haplotype study*

The families were deemed unrelated due to the absence of common surnames and connections among them. No phenocopies were allowed. The markers were chosen using the University of California Santa Cruz database (<http://genome.ucsc.edu/>), human assembly GRCh37, as previously described [S2]. The physical distances of the markers for haplotype analysis were derived from the Marshfield Comprehensive human genetic maps (<https://www.biostat.wisc.edu/~kbroman/publications/mfdmaps/>).

**TABLE S1 | Variants identified in this study**

| **Mutation (cDNA)** | **Location** | **Protein domain** | **Effect on protein** | **Mutation type** | **Variants** |
| --- | --- | --- | --- | --- | --- |
|
| c.458delT | Ex 2 | MIT | p.Asn153Metfs*8 | Small deletion | No match in dbSNP v157 |
| c.734C>G | Ex 5 | --- | p.Ser245* | Nonsense | rs1553314896 |
| c.867_868delTA | Ex 5 | MTBD | p.His289Glnfs*8 | Small deletion | rs1558323659 |
| c.906delT | Ex 6 | MTBD | p.Thr303Profs*12 | Small deletion | No match in dbSNP v157 |
| c.943_944delinsG | Ex 6 | MTBD | p. Lys315Glyfs*14 | Insertion–deletion | This study |
| c.1004+2T>G | Int 6 | --- | Aberrant splicing | Splice site | rs1553315240 |
| c.1031T>A | Ex 7 | AAA | p.Ile344Lys | Missense | rs121908513 |
| c.1054C>T | Ex 7 | AAA | p.Gln352* | Nonsense | rs1678826101 |
| c.1055A>C | Ex 7 | AAA | p.Gln352Pro | Missense | No match in dbSNP v157 |
| c.1098+1G>T | Int 7 | AAA | Aberrant splicing | Splice site | rs1377020559 |
| c.1108G>A | Ex 8 | AAA | p.Gly370Arg | Missense | No match in dbSNP v157 |
| c.1126A>G | Ex 8 | AAA | p.Arg376Gly | Missense | This study |
| c.1127G>T | Ex 8 | AAA | p.Arg376Ile | Missense | This study |
| c.1172T>C | Ex 8 | AAA | p.Leu391Pro | Missense | rs1553316845 |
| c.1173+1G>A | Int 8 | AAA | Aberrant splicing | Splice site | rs1060502226 |
| c.1174-2A>C | Int 8 | AAA | Aberrant splicing | Splice site | rs1553317018 |
| c.1196C>T | Ex 9 | AAA | p.Ser399Leu | Missense | rs1553317025 |
| c.1244insA | Ex 9 | AAA | p.Tyr415* | Small insertion | No match in dbSNP v157 |
| c.1340_1341insT | Ex 11 | AAA | p.Leu447Phefs*3 | Small insertion | No match in dbSNP v157 |
| c.1355_1357delAAG | Ex 11 | AAA | p.Glu452del | Small deletion | No match in dbSNP v157 |
| c.1378C>T | Ex 11 | AAA | p.Arg460Cys | Missense | rs878854990 |
| c.1382T>G | Ex 11 | AAA | p.Leu461Arg | Missense | This study |
| c.1417C>T | Ex 12 | AAA | p.Gln473* | Nonsense | rs757130394 |
| c.1493G>A | Ex 12 | AAA | p.Arg498Lys | Missense | rs2148754411 |
| c.1493+1G>A | Int 12 | AAA | Aberrant splicing | Splice site | rs1553318351 |
| c.1495C>T | Ex 13 | AAA | p.Arg499Cys | Missense | rs121908511 |
| c.1496G>A | Ex 13 | AAA | p.Arg499His | Missense | rs878854991 |
| c.1507C>T | Ex 13 | AAA | p.Arg503Trp | Missense | rs864622162 |
| c.1508G>T | Ex 13 | AAA | p.Arg503Leu | Missense | rs1553319087 |
| c.1534_1536del | Ex 13 | AAA | p.Glu512del | Small deletion | rs1558339948 |
| c.1536+1G>T | Int 13 | AAA | Aberrant splicing | Splice site | rs1553319095 |
| c.1540A>G | Ex 14 | AAA | p.Arg514Gly | Missense | rs1553319286 |
| c.1543_1545delCTA | Ex 14 | AAA | p.Leu515del | Small deletion | No match in dbSNP v157 |
| c.1553C>T | Ex 14 | AAA | p.Leu518Pro | Missense | No match in dbSNP v157 |
| c.1625A>G | Ex 15 | AAA | p.Asp542Gly | Missense | rs142053576 |
| c.1649C>T | Ex 15 | AAA | p.Thr550Ile | Missense | rs1553319537 |
| c.1663G>T | Ex 15 | AAA | p.Asp555Tyr | Missense | No match in dbSNP v157 |
| c.1679C>T | Ex 15 | AAA | p.Pro560Leu | Missense | No match in dbSNP v157 |
| c.1684C>T | Ex 15 | AAA | p.Arg562* | Nonsense | rs121908518 |
| c.1714_1715 delAT | Ex 16 | AAA | p.Met572Valfs*3 | Small deletion | No match in dbSNP v157 |
| c.1728+1G>A | Int 16 | AAA | Aberrant splicing | Splice site | rs587777754 |
| c.1729-1G>C | Int 16 | AAA | Aberrant splicing | Splice site | rs1064793976 |
| c.1741C>T | Ex 17 | AAA | p.Arg581* | Nonsense | rs778023258 |
| c.1849T>G | Ex 17 | --- | p.*617Gluext* 47 | No-stop change | rs1553321270 |
| del exons 1-17 | Ex 1-17 |  | Absence of allele | Large deletion | - |
| del exons 2-17 | Ex 2-17 |  | Unknown | Large deletion | - |
| del exons 6-7 | Ex 6-7 |  | Unknown | Large deletion | - |
| del exon 9 | Ex 9 |  | p.Ala492_Tyr415del | Large deletion | - |
| del exons 10-12 | Ex 10-12 |  | Unknown | Large deletion | - |
| del exons 14-17 | Ex 14-17 |  | Unknown | Large deletion | - |
| del exon 17 | Ex 17 |  | Unknown | Large deletion | - |
| dup exons 10–12 | Ex 10–12 |  | Unknown | Large duplication | - |

The table summarizes the pathogenetic variants identified in this study (based on NM_014946.4), their location, the affected protein domain, and the type. Variants already described and present in dbSNP database have been reported, while ~~N~~novel variants have been marked (This Study).

**Table S2 |** Bioinformatic analysis of the new variants identified

| **Variant** | **Effect on translation** | **CAAD** | **SIFT** | | **Polyphen 2**  **(HumVar)** | | **AlphaMissense** | |
| --- | --- | --- | --- | --- | --- | --- | --- | --- |
|  |  | PHRED Score* | Prediction | Score^#^ | Prediction | Score^†^ | Prediction | Score^§^ |
| p. Lys315Glyfs*14 | frameshift | No match | - | - | - | - | - | - |
| p.Arg376Gly | missense | 26.60 | Damaging | 0.003 | Probably Damaging | 0.977 | Likely Pathogenic | 0.795 |
| p.Arg376Ile | missense | 32.00 | Damaging | 0.002 | Probably Damaging | 0.953 | Likely Pathogenic | 0.95 |
| p.Leu461Arg | missense | 29.40 | Damaging | 0.0 | Probably Damaging | 0.999 | Likely Pathogenic | 0.995 |

*CADD, Combined Annotation Dependent Depletion, version 1.7: high PHRED scores are associated with a more likelihood to be deleterious. For instance, a score of 20 indicates the variant is in the top 1% most deleterious

^#^Threshold < 0.05 for Damaging

^†^ Thresholds ≥ 0.447|0.909 for Possibly and Probably Damaging

^§^ Thresholds 0.34|0.564 for Likely Benign, Ambiguous, Likely Pathogenic

**Table S3 |** Clinical findings of the patients of family ITA-133

|  | **I:2** | **II:1** | **II:3** | **III:1** | **III:3** |
| --- | --- | --- | --- | --- | --- |
| **Sex** | F | M | F | M | M |
| **Age at onset, years*** | 53 | 41 | 39 | 39 | 38 |
| **Age at examination** | 82 | 64 | 61 | 51 | 49 |
| **Disease Duration** | 29 | 23 | 22 | 12 | 11 |
| **Disability stage^†^** | 4 | 3 | 2 | 2 | 3 |
| **SPRS^‡^** | 35 | 27 | 26 | 16 | 22 |
| **LLH^§^** | + | + | + | + | + |
| **LLS^¶^** | +++ | ++ | + | + | ++ |
| **LLW^#^** | ++ | + | + | - | + |
| **Babinski sign** | B | B | U | U | B |
| **Sphincter disturbances** | + | + | + | - | - |
| ***Pes cavus*** | B | B | B | U | B |
| **Dementia** | + | AP | + | + | + |
| **Age at onset of cognitive dysfunction** | 62 | 52 | 50 | 47 | 45 |
| **Cognitive dysfunction duration** | 20 | 12 | 11 | 4 | 4 |
| **CSF amyloid ratio test^** | 0.022 | 0.028 | 0.033 | 0.031 | 0.033 |
| **CSF p-tau 181 test^$^** | 36.4 | 34.1 | 29.6 | 31.3 | 29.9 |
| **MMSE^**^** | 8 | 9 | 14 | 12 | 14 |
| **CAMCOG^††^** | 31 | 33 | 48 | 44 | 47 |
| **Intellectual disability** | - | - | - | - | - |
| **Brain MRI cerebral atrophy** | + | + | + | + | + |
| **Degree of cerebral atrophy^‡‡^** | 3 | 4 | 1 | 1 | 1 |
| **Brain FLAIR^§§^ MRI white matter abnormalities** | - | - | - | - | - |
| **Brain FLAIR MRI “Ears of the Lynx Sign”** | - | - | - | - | - |
| **MRI spinal atrophy** | + | + | + | + | + |
| **Degree of spinal atrophy^¶¶^** | 4 | 3 | 2 | 2 | 3 |
| **Amyloid PET imaging^##^** | 3 (81) | 3 (75) | 3 (51) | 3 (56) | 3 (53) |

M = male; F = female; U = unilateral; B = bilateral; AP = autopsy proven; + and – indicate the presence or absence of a feature, respectively; *****Age at onset was calculated approximately as the time when difficulty walking first appeared in the affected individuals; **^†^**Disability stages: 1, no mobility problems or slight stiffness of the legs; 2, moderate gait stiffness; 3, problems running, but able to walk alone; 4, problems walking; 5, wheelchair-bound; **^‡^**SPRS = Spastic Paraplegia Rating Scale; **^§^**LLH, lower limbs hyperreflexia; **^¶^**LLS, lower limbs spasticity; **^#^**LLW, lower limbs weakness; ^CSF amyloid ratio test. Aβ_1-42_/Aβ_1-40_ ratio of 0.073 and above, normal; Aβ_1-42_/Aβ_1-40_ ratio between 0.059 and 0.072, likely positive; Aβ_1-42_/Aβ_1-40_ ratio of 0.058 and below, positive; ^$^CSF p-tau 181 test. Cognitive impairment (≥50.0 pg/mL); **^**^**MMSE = Mini Mentale State Examination. Severe (≤9 points), moderate (10–18 points), or mild (19–23 points) cognitive impairment; **^††^**CAMCOG = Cambridge Cognitive Evaluation battery. Severe (≤33 points), moderate (34–63 points), or mild (64–79 points) cognitive deficit; **^‡‡^**Degree of cerebral atrophy: 1, normal; 2, mild; 3, moderate; 4, severe; **^§§^**FLAIR = Fluid Attenuated Inversion Recovery;**^¶¶^**Degree of spinal atrophy: 1, normal; 2, mild; 3, moderate; 4, severe; ^##^[18F]flutemetamol retention in the cortical regions: 0, no retention (< 10 Centiloids, CL); 1, mild retention (10-30 CL); 2, moderate retention (30-50 CL); 3, severe retention (> 50 CL).

**Table S4 |** Clinical findings of the index patients of families BRA-36, JAP-28, and JAP-31

|  | **BRA-36 II:1** | **JAP-28 II:1** | **JAP-28 II:3** |
| --- | --- | --- | --- |
| **Sex** | M | F | M |
| **Age at onset, years*** | 43 | 48 | 45 |
| **Age at examination** | 71 | 68 | 70 |
| **Disease Duration** | 28 | 23 | 25 |
| **Disability stage^†^** | 4 | 3 | 3 |
| **SPRS^‡^** | 38 | 26 | 27 |
| **LLH^§^** | + | + | + |
| **LLS^¶^** | +++ | ++ | ++ |
| **LLW^#^** | ++ | + | + |
| **Babinski sign** | B | B | B |
| **Sphincter disturbances** | + | + | + |
| ***Pes cavus*** | B | B | B |
| **Dementia** | AP | AP | AP |
| **Age at onset of cognitive dysfunction** | 57 | 53 | 54 |
| **Cognitive dysfunction duration** | 14 | 15 | 16 |
| **CSF amyloid ratio test^** | 0.021 | 0.028 | 0.026 |
| **CSF p-tau 181 test^$^** | 33.5 | 29.9 | 30.6 |
| **MMSE^**^** | 9 | 12 | 11 |
| **CAMCOG^††^** | 29 | 39 | 37 |
| **Intellectual disability** | - | - | - |
| **Brain MRI cerebral atrophy** | + | + | + |
| **Degree of cerebral atrophy^‡‡^** | 4 | 4 | 4 |
| **Brain FLAIR^§§^ MRI white matter abnormalities** | - | - | - |
| **Brain FLAIR MRI “Ears of the Lynx Sign”** | - | - | - |
| **MRI spinal atrophy** | + | + | + |
| **Degree of spinal atrophy^¶¶^** | 4 | 3 | 3 |
| **Amyloid PET imaging^##^** | 3 (80) | 3 (69) | 3 (73) |

M = male; F = female; U = unilateral; B = bilateral; AP = autopsy proven; + and – indicate the presence or absence of a feature, respectively; *****Age at onset was calculated approximately as the time when difficulty walking first appeared in the affected individuals; **^†^**Disability stages: 1, no mobility problems or slight stiffness of the legs; 2, moderate gait stiffness; 3, problems running, but able to walk alone; 4, problems walking; 5, wheelchair-bound; **^‡^**SPRS = Spastic Paraplegia Rating Scale; **^§^**LLH, lower limbs hyperreflexia; **^¶^**LLS, lower limbs spasticity; **^#^**LLW, lower limbs weakness; ^CSF amyloid ratio test. Aβ_1-42_/Aβ_1-40_ ratio of 0.073 and above, normal; Aβ_1-42_/Aβ_1-40_ ratio between 0.059 and 0.072, likely positive; Aβ_1-42_/Aβ_1-40_ ratio of 0.058 and below, positive; ^$^CSF p-tau 181 test. Cognitive impairment (≥50.0 pg/mL); **^**^**MMSE = Mini Mentale State Examination. Severe (≤9 points), moderate (10–18 points), or mild (19–23 points) cognitive impairment; **^††^**CAMCOG = Cambridge Cognitive Evaluation battery. Severe (≤33 points), moderate (34–63 points), or mild (64–79 points) cognitive deficit; **^‡‡^**Degree of cerebral atrophy: 1, normal; 2, mild; 3, moderate; 4, severe; **^§§^**FLAIR = Fluid Attenuated Inversion Recovery;**^¶¶^**Degree of spinal atrophy: 1, normal; 2, mild; 3, moderate; 4, severe; ^##^[18F]flutemetamol retention in the cortical regions: 0, no retention (< 10 Centiloids, CL); 1, mild retention (10-30 CL); 2, moderate retention (30-50 CL); 3, severe retention (> 50 CL).

**Table S5 |** Clinical findings of the patients of family BRA-56

|  | **I:1** | **II:1** | **II:2** | **II:4** | **III:1** | **III:3** |
| --- | --- | --- | --- | --- | --- | --- |
| **Sex** | M | F | M | F | M | F |
| **Age at onset*** | 41 | 48 | 53 | 56 | 39 | 35 |
| **Age at examination** | 83 | 64 | 62 | 59 | 47 | 49 |
| **Disease duration** | 42 | 16 | 9 | 3 | 8 | 14 |
| **Disability stage^†^** | 4 | 3 | 2 | 1 | 2 | 3 |
| **SPRS^‡^** | 41 | 32 | 21 | 12 | 16 | 27 |
| **LLH^§^** | + | + | + | + | + | + |
| **LLS^¶^** | +++ | ++ | + | + | + | ++ |
| **LLW^#^** | +++ | ++ | + | + | + | + |
| **Babinski sign** | B | U | U | U | U | U |
| **Sphincter disturbances** | + | + | - | - | - | + |
| ***Pes cavus*** | B | B | U | - | U | B |
| **Intellectual disability^**^** | +++ | ++ | + | + | + | ++ |
| **WAIS-R IQ^††^** | 33 | 47 | 62 | 65 | 66 | 46 |
| **Dementia** | - | - | - | - | - | - |
| **Brain MRI thin *corpus callosum*** | + | + | + | + | + | + |
| **Brain MRI cerebral atrophy** | - | - | - | - | - | - |
| **Brain FLAIR^‡‡^ MRI white matter abnormalities** | - | - | - | - | - | - |
| **Brain FLAIR MRI “Ears of the Lynx Sign”** | - | - | - | - | - | - |
| **MRI spinal atrophy** | + | + | + | + | + | + |
| **Degree of spinal atrophy^§§^** | 2 | 2 | 3 | 3 | 3 | 2 |

M = male; F = female; U = unilateral; B = bilateral; + and – indicate the presence or absence of a feature, respectively; *****Age at onset was calculated approximately as the time when difficulty walking first appeared in the affected individuals; **^†^**Disability stages: 1, no mobility problems or slight stiffness of the legs; 2, moderate gait stiffness; 3, problems running, but able to walk alone; 4, problems walking; 5, wheelchair-bound; **^‡^**SPRS = Spastic Paraplegia Rating Scale; **^§^**LLH, lower limbs hyperreflexia; **^¶^**LLS, lower limbs spasticity; **^#^**LLW, lower limbs weakness; **^**^**Intellectual disability levels: +, mild; ++, moderate; +++, severe; **^††^**WAIS-R IQ = Wechsler Adult Intelligence Scale - Revised Intelligence Quotient; **^‡‡^**FLAIR = Fluid Attenuated Inversion Recovery; **^§§^**Degree of spinal atrophy: 1, normal; 2, mild; 3, moderate; 4, severe.

**Supplementary References**

S1. M. M. Li, M. Datto, E. J. Duncavage, et al., “Standards and guidelines for the interpretation and reporting of sequence variants in cancer: a joint consensus recommendation of the Association for Molecular Pathology, American Society of Clinical Oncology, and College of American Pathologists,” *Journal of Molecular Diagnostics* 19, no. 1 (2017): 4–23.

S2. J. von Salomé, T. Liu, M. Keihäs, et al., “Haplotype analysis suggests that the *MLH1* c.2059C>T mutation is a Swedish founder mutation,” *Familial Cancer* 17 (2018): 531–537.
